# Suzanne Verdonschot, Marloes de Jong, Paul Keursten: Innovationen im Hier und Jetzt – Eine Toolbox mit neuen Ideen und ungewöhnlichen Ansätzen zum Ausprobieren

**DOI:** 10.3205/zma000991

**Published:** 2015-11-16

**Authors:** Sigrid Harendza

**Affiliations:** 1 Universitätsklinikum Hamburg-Eppendorf, III. Medizinische Klinik, Hamburg, Deutschland

## Recension

**Figure 1:**
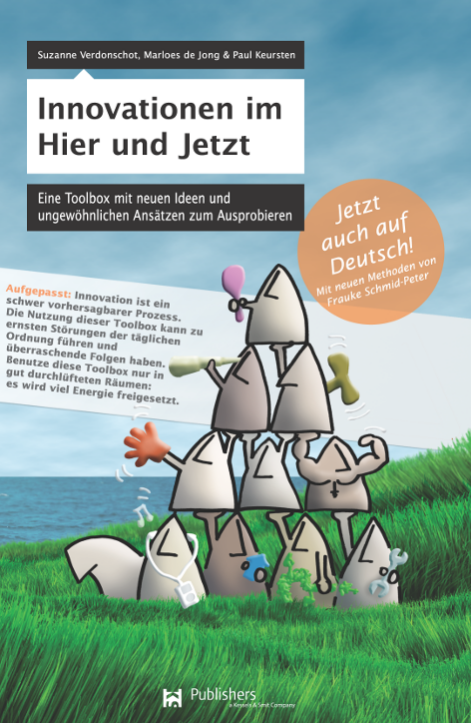
Cover

To say it straight away: the toolbox is not a book (see figure 1 (Fig. 1)), rather a game. But not really – rather a tool kit with instructions in an appealing box. It originated from a research project. But first things first.

As in medical education, innovation processes play an important role in many other fields and the involved stakeholders struggle with similar problems. However, such difficulties in development processes can also be considered as tasks which have to be solved successfully without wrecking one’s own nerves. The handbook enclosed in the toolbox reports the investigation of such processes which can be successfully worked on with proper “project management”. Not only does it provide explanations, but it also offers tools printed on handy cards which can be directly used in one’s own workshop. Hence, it is very useful for everyone who wishes to manage group processes that do not always run smoothly (e.g. curriculum development).

In the first chapter of the handbook, innovations are described as a mutual learning process that depends on the different perspectives. So called “innovation practices” are the focus of this chapter, i.e. groups of people who want to work jointly on a difficult task in practice. By means of investigations of different innovation practices from different work and life areas the authors developed a model for the design process of breakthroughs to facilitate innovations. In addition, they identified factors in several studies which influence the design process. Being aware of these relationships they developed 11 design principles in the second chapter of this handbook which can serve as guidelines for individual work in innovation processes in practice.

These design principles sound very intuitive and pragmatic, for instance: „Draft an urgent and fascinating question” or “Establish unfamiliar contacts between people with different expertise” or “Work on the basis of mutual attraction”. All design principles are displayed according to the same principle. Initially, the mode of operation of the principle is explained, afterwards, the principle is exemplified further with a practical example, and finally, a guideline is given how to use this principle in one’s own practice. Every design principle is prefaced by a motto and a cartoon which help to memorize the principle easily. An abbreviated version of every design principle can be found in the toolbox printed on a DIN A6 card including important tips to be used in practice.

The toolbox also contains a set of 27 DIN A5 cards, so called cards with methods, which described possible methods to transfer the different design principles suggested in the handbook into practice. These cards with methods include precise instructions to use the respective method in an appropriate situation. At the same time they leave a wide scope to the user to experiment with the methods. Since not every method works for every innovation practice and every facilitator in a similar fashion, the toolbox is complemented by an internet page (http://www.innovationenimhierundjetzt.de) where one can use a self-assessment test to evaluate which methods fits one’s personality and mode of working. At the same time, further worksheets can be found for practical use.

The toolbox is made perfect with a second set of 14 DIN A6 cards which contain exemplary situations that can be supportively used to enter the mode of operation with one’s own innovation. Chapter 3 of the handbook being entitled “To work with the whole set of principles” provides further support. The design process model from the first chapter is taken up again and used to explain the five steps within the planning process. On the basis of the design principles and the cards with methods provided in the toolbox these steps can be made and tested from the identification of a problem within one’s own design process via the planning of a methodical intervention to the evaluation of the effect of this innovation. The list of references in the back of the handbook provides the evidence for the methods contained in the toolbox.

For everyone, who is stuck in development processes, who fights resistance in planning groups or who would like to start an innovation process in the right way straight from the beginning, it is a blessing, that this toolbox, which was developed in the Netherland in 2009, is now released in German language. The pragmatic sets of cards allow for an immediate use of evidence-based methods in one’s own practice and are well suited as little memory aids in practical workshop use also for newcomers in the field of project management.

## Competing interests

The author declares that she has no competing interests.

